# Substrate specificity and transport mechanism of amino-acid transceptor Slimfast from *Aedes aegypti*

**DOI:** 10.1038/ncomms9546

**Published:** 2015-10-09

**Authors:** Dmitri Y. Boudko, Hitoshi Tsujimoto, Stacy D. Rodriguez, Ella A. Meleshkevitch, David P. Price, Lisa L. Drake, Immo A. Hansen

**Affiliations:** 1 Department of Physiology and Biophysics, Rosalind Franklin University, North Chicago, Illinois 60064, USA; 2 Department of Biology, New Mexico State University, Las Cruces, New Mexico 88003, USA; 3 Molecular Biology Program, New Mexico State University, Las Cruces, New Mexico 88003, USA; 4 Institute for Applied Biosciences, New Mexico State University, Las Cruces, New Mexico 88003, USA

## Abstract

Anautogenous mosquitoes depend on vertebrate blood as nutrient source for their eggs. A highly efficient set of membrane transporters mediates the massive movement of nutrient amino acids between mosquito tissues after a blood meal. Here we report the characterization of the amino-acid transporter Slimfast (Slif) from the yellow-fever mosquito *Aedes aegypti* using codon-optimized heterologous expression. Slif is a well-known component of the target-of-rapamycin signalling pathway and fat body nutrient sensor, but its substrate specificity and transport mechanism were unknown. We found that Slif transports essential cationic and neutral amino acids with preference for arginine. It has an unusual dual-affinity mechanism with only the high affinity being Na^+^ dependent. Tissue-specific expression and blood meal-dependent regulation of Slif are consistent with conveyance of essential amino acids from gut to fat body. Slif represents a novel transport system and type of transceptor for sensing and transporting essential amino acids during mosquito reproduction.

## Abstract (web summary)

Anautogenous mosquitoes need to obtain essential amino acids from a blood meal for reproduction. Here, the authors examine the amino acid transporter Slimfast from the yellow-fever mosquito and describe both its specificity and mechanism of action.

## Graphical abstract

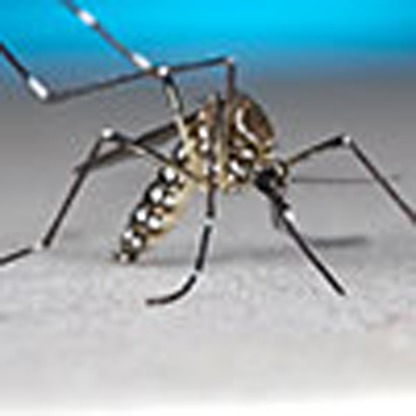

A principal feature in metazoan evolution is the loss of pathways for the biosynthesis of a group of nine essential amino acids (AAs)^1^. Concurrently, metazoans acquired effective mechanisms for the uptake of these and other AAs from their food, their distribution within the body, and the monitoring of intracellular and extracellular AA concentrations^2^,^3^.

A blood meal (BM) taken from a vertebrate host is the source of essential AAs for egg development in anautogenous mosquitoes^4^. Post blood meal (PBM) females mosquitoes undergo complex changes in tissue-specific gene expression in order to transfer nutrient AAs from the digested meal to the developing oocytes, a process called vitellogenesis^5^,^6^. Vitellogenesis is triggered upon the initial detection of a surge of free AAs in the mosquito circulation^7^,^8^ and involves coordinated hormonal and nutritional regulation of enzymatic and transport processes in midgut (GT), fat body (FB) and ovaries (OVs)^8^,^9^,^10^. The three subsequent key mechanisms of this process are: BM digestion in the GT, synthesis and secretion of yolk protein precursors by the FB, and receptor-mediated uptake and deposition of yolk protein precursors and other nutrient reserves in developing oocytes.

Up to 19% of the total ingested AAs are used for the synthesis of soluble yolk proteins while the rest is largely used for energy production^11^,^12^,^13^. The digested AAs are transported across the apical and the basolateral membranes of the gut epithelium into the haemolymph and subsequently across the plasma membrane of FB cells. The yolk proteins synthesized by the FB are secreted into the haemolymph and are absorbed by the developing oocytes via receptor-mediated endocytosis^14^,^15^. Evidently, the membrane transport of free AAs is also necessary to supply a surge of protein synthesis in the OVs PBM^16^. Hence, the rapid redistribution of nutrient AAs during mosquito vitellogenesis requires highly regulated and efficient transport mechanisms. However, the molecular properties and regulation of such mechanisms are only partly explored.

To date, only a few transporters that mediate uptake of nutrient AAs in mosquitoes have been characterized^2^,^17^,^18^. Most of these are expressed in the larval stage and their role and significance in adult metabolism remains enigmatic. The aquatic larval stage of mosquitoes substantially differs from the terrestrial reproductive stage in terms of nutrient availability and metabolic demand^2^,^19^.

In previous work, we have identified and characterized several AA transporters from the Solute Carrier Family 7 (SLC7) of *Ae. aegypti*, the yellow-fever mosquito. The SLC7 family includes two subgroups: the cationic AA transporters (CATs) and the heterodimeric AA transporters (HATs)^20^. RNA interference-mediated knockdown of 6 of the 11 members of the SLC7 family reduced target of rapamycin (TOR)-mediated nutrient signalling in the mosquito FB, which resulted in limited egg production^21^,^22^. We cloned and characterized a first mosquito CAT, *Aa*CAT1, and found that it is a Na^+^-independent transporter with a unique selectivity to L-histidine^23^. The *Aa*CAT1 paralogue, *Aa*Slif (*=*CAT3; GenBank accession #: XP_001662274) is closely related to fruit fly slimfast (slif). Characterization of *slif* homologues in mosquito is of high interest as it may function as a mechanism combining transporter and receptor properties (transceptor). *Drosophila* slif is involved in signalling AA-availability in the metabolic regulation of fly growth^24^ and behavioural control of feeding arousal^25^. Both these functions comprise appealing targets for vector control. However, transport function of slif was unknown and could not be extrapolated from mammalian paralogues and insect CATs because of significant functional divergence of these transporters.

Here we describe the tissue-specific expression along with the biochemical–biophysical properties of *Aa*Slif (=*Aa*CAT3). Unexpectedly, our study revealed an unusual set of biophysical properties of this transporter including: dual-affinity for cationic and neutral AAs, lack of L-isomer selectivity, and a significant Na^+^-dependent component necessary for its high-affinity transport mode. These findings expand our current knowledge of transport functions and biological significance of the CAT-SLC7 mechanisms. The present studies also describe properties of a new AA transceptor with critical roles in nutrition and reproduction of an important disease vector.

## Results

### Cloning of *Aa*Slif and genomic structure of a CAT cluster

To determine the *Aa*Slif cDNA sequence, we used Rapid Amplification of cDNA Ends (5′ RACE). We found an additional intron and an exon at the 5′-end of the transcript that was absent in the current annotation of the *Ae. aegypti* genome (VectorBase AAEL012131; see Supplementary Fig. 1). The *Aa*Slif gene consists of five exons and four introns, which are spliced to an 1,881-nucleotide-long open reading frame (ORF) encoding a 626-AA protein. When compared with the published genome sequence^26^, the *Aa*Slif ORF contains 21 synonymous SNPs, which likely represent differences between the Liverpool (Genome sequence in VectorBase) and the Rockefeller strains (this study) of *Ae. aegypti*. The gene is located on the reverse strand of the supercontig 1.658 (Supplementary Fig. 1A). Two other genes of the *Aa*CAT-SLC7 subfamily (*Aa*CAT1 and *Aa*CAT2) are localized in a proximal reverse cluster downstream from *Aa*Slif. The fourth gene of the *Aa*CAT-SLC7 subfamily AAEL012129 is found in the forward strand and encodes the complete ORF of *Aa*CAT4 (Supplementary Fig. 1A). *Aa*CAT2 (AAEL012133) was only partly predicted in the current annotation of *Aedes* genome (Supplementary Fig. 1, brown splicing pattern). Our *in silico* analysis revealed a splicing scheme that is also supported by the gene expression pattern found in studies of the mosquito transcriptome (Supplementary Fig. 1, red+brown splicing pattern and magenta bars, respectively; based on the data from VectorBase).

### Selection and conservation of the SBM in the Slif mechanism

In a previous phylogenetic study, we found that *D. melanogaster slif* (*Dm*Silf) forms a phylogenetic cluster with two putative orthologues from *Ae. aegypti*, *Aa*CAT1 and *Aa*Slif^23^. Pairwise identity between *Dm*Slif and *Aa*CAT1 is 56.9%; between the *Dm*Slif and *Aa*Slif 58.6 %, with 337 and 367 identical sites, respectively. To identify structure-function conservation among members of this cluster, we aligned the AA sequences of *Dm*Slif orthologues from selected insect representatives against sequences and structural motifs of the recently crystalized prokaryotic relatives of the SLC7 family: the *Methanocaldococcus jannaschii* ApcT^27^ and *Escherichia coli* AdiC^28^ (Supplementary Fig. 1B). In spite of only moderate pairwise identity, the prokaryotic and eukaryotic transporters aligned well in a region between the first and tenth transmembrane domain (TMD). The prokaryotic TMDs 11 and 12 aligned well to insect TMDs 11–12 as well as 13–14 with similar homology patterns (Supplementary Fig. 1B). The possibility of an alternative alignment supports the idea that the two C-terminal TMDs were duplicated in an universal CAT ancestor^23^.

**Figure 1:**
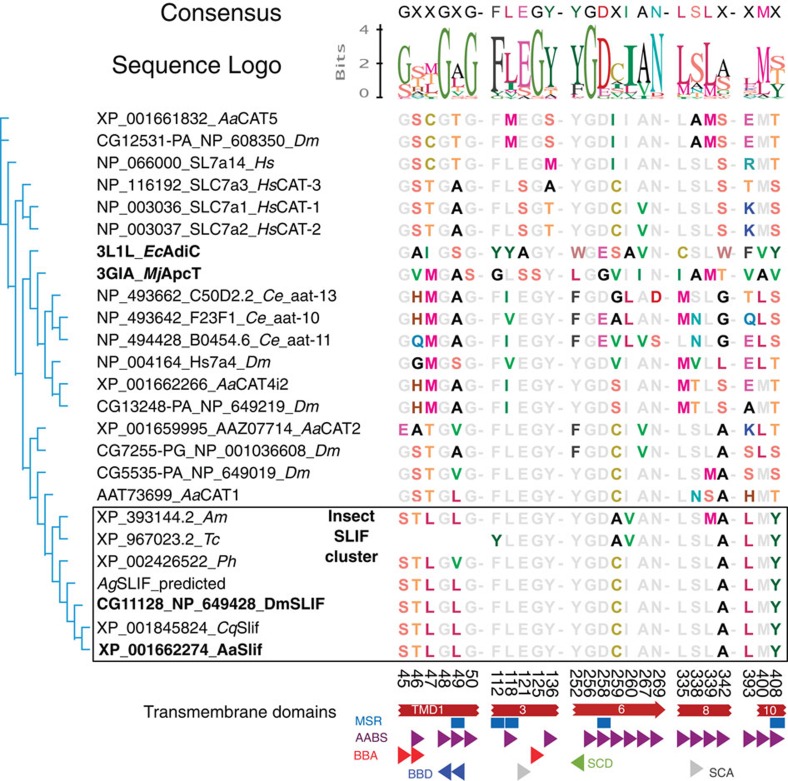
Bioinformatics analysis of the *Aa*Slif substrate-binding motif (SBM). Shown are a neighbour-joining consensus tree and an alignment of the putative SBM residues of selected CAT-SLC7 members. Putative Slif orthologues (outlined box) form a phylogenetic cluster with exactly one orthologue per selected insect genome and also show strong conservation of the SBM. The names of *Drosophila* and *Aedes* slif orthologues and their prokaryotic homologues used for the identification and alignment of SBMs are highlighted by bold font. Coloured and grey fonts depict variable and significantly conserved residues, respectively. The aligned numbers indicate the amino-acid positions in the *Aa*Slif protein sequence. The partial SBM of the *Tribolium* Slif is due to its incomplete annotation. The coloured shapes depict specific putative components of the SBMs abbreviated as: AABS, amino acid-binding sites; BBA, backbone acceptor; BBD, backbone donor; MSR, mutation sensitive residues; SCA, side chain acceptor; SCD, side chain donor. Species abbreviations are: Aa*, Aedes aegypti*; Ce, *Caenorhabditis elegans*; Cq, *Culex quinquefasciatus*; Hs, *Homo sapiens*; Ph, *Pediculus humanus*; Tc, *Tribolium castaneum*.

Figure 1 shows the alignment pattern of a putative substrate-binding motif (SBM) interpolated from the *Ec*AdiC three-dimensional (3D) structure. It reveals both highly conserved and variable sites. Importantly, it defines that the predicted SBM residues are identical among dipteran *slif* orthologues and also are strongly conserved in the flour beetle (*Tribolium castaneum*), the body louse (*Pediculus humanus corporis*) and the honey bee (*Apis mellifera*) orthologues.

### Heterologous expression and electrophysiological characterization

The heterologous expression of the original *Aa*Slif transcript (GenBank accession #: KM593906) in *Xenopus laevis* oocyte resulted in a significant increase of Arg-induced currents compared with water-injected control oocytes (*I*=10.2±5.8 nA for 10 mM L-Arg; *n*>3, *P*=0.014; *t*-test). However, such a signal was considered too weak for an electrophysiological characterization of the transport mechanism.

To improve *Aa*Slif expression, we ordered synonymous optimized synthetic variants of *Aa*Slif with a *Xenopus*-codon usage (synthesized by Genwiz Inc.) and cloned them in the pXOOM expression vector. Two plasmids: *Aa*Slif_co_-pXOOM (GenBank accession #: KM593906) and *Aa*slif_co_eGFP-pXOOM (GenBank accession #: KM593907) both with the *Aa*Slif ORF and the latter also fused to enhanced green fluorescent protein (eGFP) at the C-terminus, were tested.

**Figure 2:**
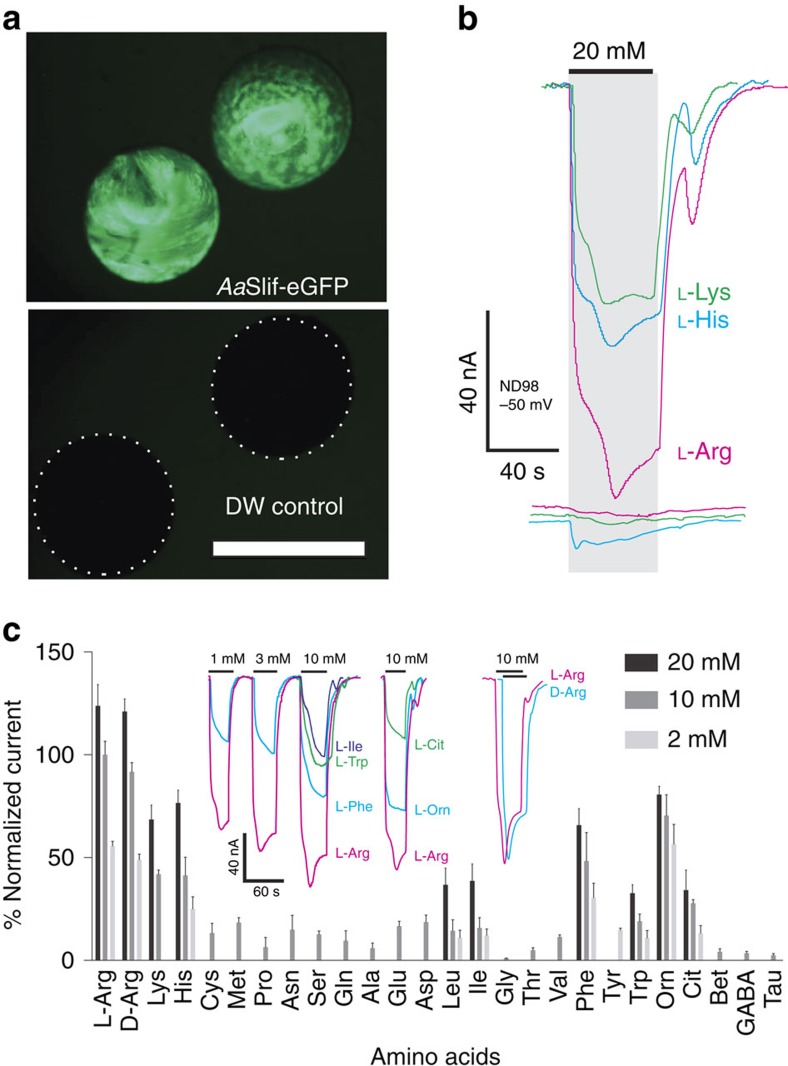
Functional expression and AA-specificity profile of *Aa*Slif. (**a**) Functional expression of codon-optimized *Aa*Slif-eGFP fusion protein showed bright fluorescence compared with control deionized water-injected oocytes (bottom panel, white dashed outlines indicate position of control oocytes that are almost invisible with identical settings of the GFP-specific filter cube (Nikon 49583; EN GFP LP: Excitation Hq 470/40, Dichroic 495EM, Emission 500LP) and camera sensitivity); scale bar represents 1 mm. (**b**) The expression of *Aa*Slif correlates with large cationic AA-induced current responses. The coloured lines represent currents induced in the *Aa*Slif-eGFP-expressing oocytes (top traces) versus control oocytes (bottom traces) after application of 20 mM of L-enantiomer of Lysine (K), Histidine (H) and Arginine (R). (**c**) AA specificity profile reconstructed from AA-induced currents. The bars are mean of percent normalized current+s.d. (*n*>2 oocytes/samples per point) at 20, 10 and 2 mM concentrations of AAs. Currents were recorded using two-electrode voltage clamp. The coloured traces show representative recordings of currents induced by three different concentration of L-Arg and L-Phe, as well as 10 mM of L-Trp, L-Ile, L-Arg, L-Orn and L-Cit. Oocytes were clamped to –50 mV holding voltage in a constant flow micro-chamber perfused at ∼2-chamber volumes per second with ND98 or indicated solutions of AAs in 98 Na^+^ buffer. Currents were measured considering a steady-state current value and normalized to the mean of 10 mM L-Arg (=100%) response in each oocyte for cross-oocyte comparison. Cit, citrulline; DW, deionized water; GABA, γ-aminobutyric acid; Orn, ornithine; Tau, taurine.

The expression of *Aa*Slif_co_GFP induced bright green fluorescent protein (GFP)-specific fluorescence that was correlated with a large inward current upon application of cationic AAs in oocytes on days 4–10 after injection of cRNA (∼10 nA at 1 μM and ∼100 nA at 10 mM of L-Arg; Fig. 2a). Importantly, the expression of *Aa*Slif_co_ and *Aa*Slif_co_GFP resulted in no detectable differences in measured amplitude, saturable kinetics and voltage dependency; therefore, both variants could be used interchangeably in the analysis (Supplementary Fig. 2).

*Aa*Slif has an apparent preference for Arg (Fig. 2b; Arg>His≈Lys) without significant L-D enantiomer selectivity (Fig. 2c, current trace insert). *Aa*Slif also generated significant inward currents on applications of L-citrulline (Cit) and L-ornithine (Orn), two metabolic AAs that we tested as intermediates of the nitric oxide (NO) synthesis and L-arginine recycling cascades (Supplementary Fig. 2). In contrast, betaine, γ-aminobutyric acid and taurine induced no significant responses. Notably, *Aa*Slif also generates significant inward currents for neutral aliphatic (Ile, Leu, Met), aromatic (Phe, Trp, Tyr) and even acidic AAs (Supplementary Fig. 2).

### Substrate saturation kinetics, pH dependency and uptake

**Figure 3:**
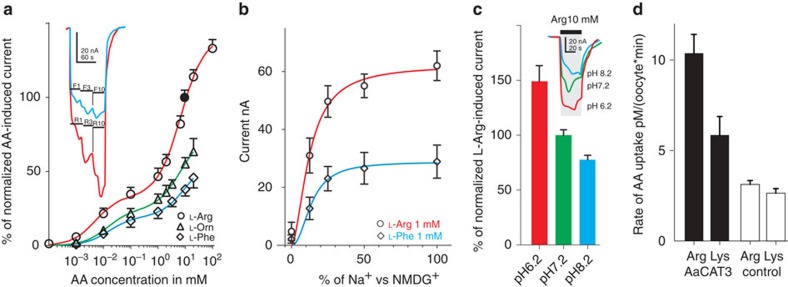
*Aa*Slif substrate saturation kinetics, pH dependency and substrate specificity. (**a**) Saturation kinetic graphs for L-Arg-, L-Phe- and L-Orn-induced currents. The insert shows the relative amplitudes of L-Arg and L-Phe-induced currents in *Aa*Slif-expressing oocytes stimulated with three different concentrations of L-Arg and L-Phe. The data were normalized between different oocytes using mean value responses to 10 mM L-Arg (Filled circle point). Data are mean±s.d. for *n*>3. (**b**) Sodium saturation kinetics for 1 mM L-Arg- and L-Phe-induced currents. Data are mean±s.d. for *n*=3. (**c**) pH dependency of 10 mM L-Arg-induced currents. Bars are mean+s.d. for *n*=3 oocyte/samples, that are significantly different *P*>0.001; *t*-test. Insert shows a scaled superposition of typical L-Arg-induced currents induced upon sequential application of L-Arg at specified pH values. (**d**) Uptake of radiolabelled cationic AAs in *Aa*Slif-expressing oocytes (black) versus control oocytes (white). Data are mean+s.d. for *n*=5 individual oocyte assays.

A substrate saturation assay revealed that *Aa*Slif has two apparent saturation points: one at ∼1 mM and second at ∼20 mM of L-Arg (Fig. 3a and Supplementary Fig. 3). The estimated *K*_d_^Hi^ and *K*_d_^Lo^ were 5.2±1.4 μM and 6.9±0.8 mM for L-Arg, 13.8±6.2 μM and 6.8±2.6 mM for L-Orn and 15.6±8.5 μM and 9.8±6.5 mM for L-Phe. Our null hypothesis of a one point saturation model was rejected with *P*<0.001, *P*=0.018 and *P*=0.034 for L-Arg, L-Phe and L-Orn, respectively (*t*-test). The amplitude of cationic and neutral AA-induced currents depended on the extracellular concentration of Na^+^, with apparent Na^+^ dissociation constant *K*_d_=12.4±1.5 and 13.8±2.2 mM and Hill constant *η*=1.8±0.6 and 2.2±1.0 at 1 mM concentration of L-Arg and L-Phe, respectively (Fig. 3b). Acidification of the extracellular solution significantly increased the amplitudes of substrate-induced inward currents (∼30–50% per pH unit; *P*<0.001 for *n*>3; *t*-test; Fig. 3c). To define the transport activity of *Aa*Slif, we performed uptake assays with selected radioactive isotope-labelled substrates. The *Aa*Slif expression increased the absorption of AAs in *Aa*Slif-recombinant oocytes compared with deionized water-injected control oocytes. These results unequivocally verified that *Aa*Slif functions as an AA transporter (Fig. 3d).

### Cation dependency of AA-induced currents in *Aa*Slif oocytes

**Figure 4:**
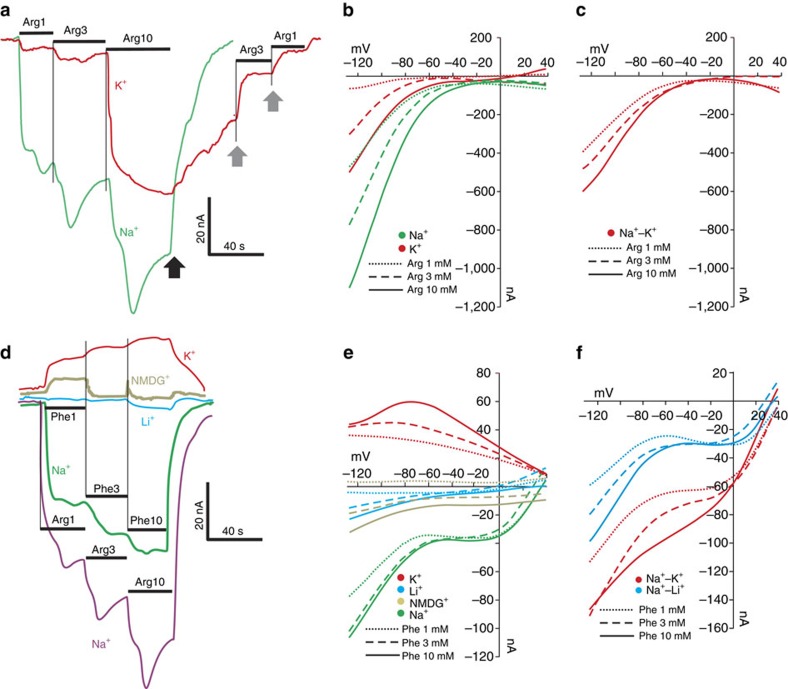
Cation dependency of AA-induced currents. (**a**) Example of substrate-induced currents in an *Aa*Slif-expressing oocyte after application of 1, 3 and 10 mM of L-Arg in 98 Na^+^ (green line) and 98 K^+^ (red line) buffers (containing identical concentrations of Cl^–^ and other ions). Black arrow indicates washing after L-Arg application with initial buffer saline. Grey arrows indicate accelerated recovery after re-application of 3 or 1 mM of L-Arg (for 98 K^+^ buffer only). (**b**) Background-subtracted *I*/*V* plots of *Aa*Slif-expressing oocytes in 98 Na^+^ and 98 K^+^ buffers with three different concentrations of L-Arg (insert). (**c**) *I*/*V* plot calculated by subtraction of the current values measured in 98 K^+^ from those in 98 Na^+^. Results show no significant dependency of current on L-Arg concentration. (**d**) L-Phe-induced currents in four different buffers. Results show that substitution of Na^+^ with Li^+^ and NMDG^+^ ions reduced substrate-induced currents, whereas the substitution with K^+^ reversed the current. (**e**) *I*/*V* plots acquired from the same oocyte as in **d** with different cation compositions, as colour-coded in insert. (**f**) *I*/*V* plots calculated by subtraction of current values measured in 98 K^+^ and 98 Li^+^ buffers from those in 98 Na^+^ buffer.

Substitution of extracellular Na^+^ by K^+^ significantly reduced the Arg-induced current (Fig. 4a) and modified voltage dependency of *Aa*Slif, shifting the inflection point towards neutral (Fig. 4b). Surprisingly, the Na^+^-K^+^ substitution resulted in much lower current induced by Arg at concentrations below the *K*_d_^Lo^ (at 1 and 3 mM of L-Arg, reduction 75±9.6% versus same in Na^+^ media, *n*>3) in comparison to the current induced by same substrate above the *K*_d_^Lo^ (at 10 mM of L-Arg, reduction 22±4.6%, *n*>3). It suggests that at low AA concentrations Arg-coupled current has large Na^+^-coupled component, while it may become largely Na^+^ independent at high concentrations of the organic substrate (Fig. 4a). The subtracted *I*/*V* plots showed additional details regarding interaction of the *Aa*Slif mechanism with Na^+^ L-Arg^+^ ions (Fig. 4c). The data suggest that Na^+^ ions have a much greater contribution in the substrate-induced current compared with Arg^+^. This effect is especially profound at transmembrane voltages below −60 mV.

We also noticed that Na^+^-K^+^ substitution delayed the recovery of the transporter after washing with 10 mM L-Arg (Fig. 4a black arrow). The reduced recovery can be rescued by extracellular administration of 3 or 1 mM L-Arg (Fig. 4a grey arrows). This indicates that *Aa*Slif can be locked in some ion conductive state upon interaction with high concentration of L-Arg at low Na^+^ or high K^+^ levels. However, a more detailed analysis of this phenomenon was beyond the scope of the characterization.

### Phenylalanine induced currents in *Aa*Slif-expressing oocytes

The L-Phe was tested as a representative of neutral aromatic substrates. Its application resulted in a substrate-induced inward current (Figs 2c and 3a,b). The Phe-induced current was strictly Na^+^-dependent. Na^+^ ions cannot be substituted by NMDG^+^ (*N*-methyl D-gluconate) or Li^+^ (Fig. 4d–f). Interestingly, the substitution of Na^+^ with K^+^ resulted in a reversion of the Phe-induced current, which implies the presence of some cationic efflux or blockage of cationic influx, for example, leak current (Fig. 4d,e). *I*/*V* plots for L-Phe with K^+^ display a characteristic nonlinearity with an inflection point close to −70 mV (Fig. 4e).

### AaSlif expression and regulation PBM

**Figure 5:**
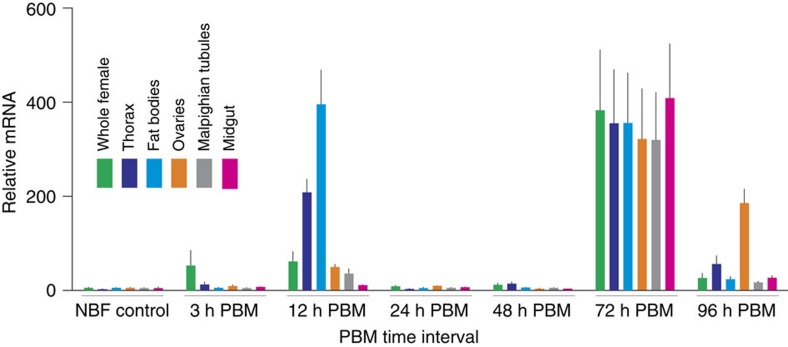
*Aa*Slif expression and regulation in selected mosquito tissues and organs. The transcript levels were determined using quantitative PCR (qPCR). The data represent relative quantities of *Aa*Slif transcript that were normalized with qPCR levels of ribosomal protein S7 (rpS7) mRNA in the same tissue sample set. Bars are means+s.e.m. for *n*=3 replicates collected from different sets of mosquitoes (analysis of variance followed by Tukey’s HSD *post hoc* test). The RNA samples were isolated from whole body and various body parts and organs of adult female mosquitoes grouped as shown by the colour-coding insert. The horizontal scale indicates group-specific conditions: non-blood fed (NBF, control), 3, 12, 24, 48, 72 and 96 h post blood meal (PBM).

To reveal spatiotemporal expression and regulation of AaSlif, we measured the accumulation/retention of its transcript in whole body, selected body parts and organ samples isolated from non-blood fed (control) and blood fed mosquito females. The samples were isolated and tested after 3, 12, 24, 48, 72 and 96-h PBM intervals. The *Aa*Slif gene showed a varied and rapidly regulated expression profile (Fig. 5 and Supplementary Fig. 4). Specifically, it was significantly upregulated in whole females at 3, 12 and 72 h PBM (∼10, 10, 60 times, respectively; *P*<0.001; two-way analysis of variance followed by Tukey’s honest significant difference (HSD) *post-hoc* tests for *n*=3 samples). However, *Aa*Slif expression declines in the whole body samples 24 and 48 h PBM close to the expression levels in the corresponding control samples. The elevated expression in 12 h PBM samples correlates with the intensified transcription of *Aa*Slif in the FBs and thorax, as well as moderately elevated transcription in the OVs and Malpighian tubules (MTs). In contrast, 72 h PBM samples showed strong overexpression across all selected body part and organs, including the mosquito gut. The level of transcript decreased in the 96-h PBM samples, except for OVs (Fig. 5).

## Discussion

The requirement for acquisition of essential AAs through blood feeding makes anautogenous *Ae. aegypti* mosquitoes an effective vector of important arboviral diseases, including dengue, yellow fever and Chikungunya^29^. BM-derived AAs are a major source of energy and building blocks during mosquito reproduction. Consequently, mosquitoes possess a highly efficient and tightly regulated system to distribute AAs between different tissues^21^. This transport system is only partly understood. In this study, we functionally expressed and characterized a mosquito orthologue of *Drosophila slif*, *Aa*Slif. This transporter represents the first characterized member of the unique insect-specific cluster of the CAT-SLC7 subfamily of AA transporters^23^,^30^ and the first characterized representative of a new transport system, SLIF.

The CAT acronym was originally coined for a group of mammalian AA transporters of the canonical y^+^ system, a Na^+^-independent transport mechanism selective for cationic L-AAs^20^,^31^. The first CAT cloned and characterized via recombinant expression in *Xenopus* oocytes was serendipitously identified as a murine leukemia virus receptor with an explicit homology to yeast AA permeases and properties of the canonical y^+^ (CAT) system^27^,^28^. Subsequent characterization of three from four existing mammalian members of the CAT-SLC7 subfamily showed similar AA selectivity patterns with some variations in affinity and tissue expression^32^. Our previous phylogenomic analysis of the CAT-SLC7 family showed that mammalian and putative insect CATs share a common ancestral root^23^. However, insect transporters formed independent clusters suggesting specificity of adaptations of CAT mechanisms in mammals and insects^23^. An important finding from the extended bioinformatics analysis of the present study is that there is only one slif orthologue per insect genome. Also, the putative SBMs of slif orthologues in fruit flies and mosquitoes are identical even though these two insect groups diverged around 0.26 Bya^33^ (Fig. 1). These evidences strongly suggest that the SBM, transport mechanism and biological functions of these transporters have undergone strong stabilizing (=purifying) selection. Another important finding is that SBMs of *slif* orthologues are distinct from the SBM of the previously characterized *Aa*CAT1 (ref. 23) and other mammalian and insect CATs (Fig. 1). These facts are consistent with the hypothesis that the slif mechanism plays a unique role in insect metabolism and lacks genetic and functional redundancy.

Previously, three mosquito CATs, *Aa*CAT1, *Aa*CAT2 and *Aa*Slif (=*Aa*CAT3), were identified as components of the FB nutrient sensor system that uses the TOR signalling pathway to activate reproductive processes in mosquito females after blood consumption^21^,^22^. In the present study, we show that this trio plus *Aa*CAT4 is physically co-localized in the mosquito genome (Supplementary Fig. 1). We also found comparable transcripts of these CATs in the transcriptomic libraries at VectorBase^34^ (Supplementary Fig. 1A, magenta pattern), supporting the notion that all four are functional genes. The tight clustering pattern suggest that CATs may be under a unified genetic control. However, considering the diverse SBMs we found in these transporters, the anticipated contributions in AA transport and signalling are clearly different. The genomic organization, together with the phylogenetic data (Fig. 1) and functional data (this study and ref. 22), suggests that the CAT cluster in the *Ae. aegypti* genome is the result of gene duplications and subsequent functional adaptation of individual CATs for a specific physiological role, which may include a substrate specialization as found in *Aa*CAT1 and *Aa*Slif.

Using codon-optimized transcripts, we achieved a significantly better expression of *Aa*Slif in *Xenopus* oocytes compared with wild-type transcript providing a unique opportunity for high-resolution electrophysiological characterization of this transporter. Chimeric fusion of *Aa*Slif with eGFP did not modify expression and functional properties of this transporter (Fig. 2a) and could be used for the visual monitoring of CAT proteins in heterologous or naïve expression systems without functional artifacts.

Our functional characterization reveals that *Aa*Slif is a high-efficiency carrier of cationic AAs with particular preference to Arg (Figs 2 and 3). It also transports urea cycle metabolites that represent products of the NO synthesis pathway^35^. These characteristics of *Aa*Slif are consistent with the generic properties of mammalian CAT-SLC7 transporters^36^. Nonetheless, the *Aa*Slif mechanism differs from the y^+^ system of previously characterized mammalian CATs^32^ and the His-specific mosquito *Aa*CAT1 (ref. 23). *Aa*Slif showed a substantially broader substrate spectrum compared with previously characterized CATs. Moreover, it equally transports L- and D-enantiomers of Arg (Fig. 2c); whereas the mammalian CATs are strictly or significantly stereo-selective^37^,^38^. It also generates significant inward currents for neutral aliphatic (Ile, Leu, Met), aromatic (Phe, Trp, Tyr) and even acidic AAs (Fig. 2c). Such currents cannot be explained by facilitated diffusion of neutral AAs or cationic/neutral AA coupled exchange mechanisms found in mammalian CAT and HAT members of the SLC7 family^23^,^30^. Such mechanisms should generate no outward currents in responses to neutral AAs. The ion substitution assay showed that the inward neutral AA-induced current is due to involvement of inorganic ions (Fig. 4d–f).

Unexpectedly, we observed complex saturation kinetics of the *Aa*Slif consistent with an unusual dual-affinity mechanism and two dissociation constants: one in low-micromolar range and other in low-millimolar ranges (Fig. 3). Notably, its substrate-induced currents were pH-dependent and correlate with the uptake of radiolabelled substrates (Fig. 3c,d, respectively). Moreover, the high-affinity mode of the *Aa*Slif interaction with L-Arg was largely Na^+^ dependent (Fig. 4a–c). The Na^+^-coupling was also associated with the neutral AA-induced inward current. This revealed a Na^+^ selectivity of the *Aa*Slif mechanism that neither Li^+^ nor NMDG^+^ ions can substitute (Fig. 4). Interestingly, substitution of Na^+^ with K^+^ resulted in an inversion of the neutral AA-induced currents that was not observed upon Na^+^ substitution with Li^+^ or NMDG^+^ (Fig. 4d–f). This indicates that K^+^ is a potent modulator of ion fluxes through *Aa*Slif, a finding that requires further experimental analysis.

We propose that the identified properties represent adaptations of *Aa*Slif for its action in an extended range of AA concentrations. At low AA availability, for example, during the state-of-arrest before the mosquito takes a BM, it acts as secondary high-affinity transporter using Na^+^ motive forces for the intracellular accumulation of AAs. In contrast, at high AA availability, for example, after a BM, it acts as a low affinity, passive uniporter facilitating transmembrane diffusion of nutrient substrates without using the electrochemical energy of ion gradients. Dual-affinity mechanisms have never previously been reported among metazoan representatives of the Major Facilitator Superfamily. However, a dual-affinity nitrate transporter (CHL1, also NRT1) has been recently reported among MFS representatives in plants^39^. When phosphorylated at Thr101, CHL1 acts as a high-affinity nitrate transporter, whereas dephosphorylated transporter has low affinity. It has been suggested that switching involves dimerization, and that dual affinity CHL1 represents adaptation for managing highly variable concentrations of critical nutrients^40^.

*Aa*Slif utilizes Na^+^ motive forces for the transport of neural AAs and also the high-affinity transport of cationic AAs. Such a coupling satisfies thermodynamic requirements for intracellular translocation of substrates from low-concentration domains. The identification of the first CAT with explicit sodium dependency is truly remarkable for the field of transport biology. Although mammalian CATs generally do not use inorganic cations for translocation of cationic AAs, some Na^+^ dependency has been reported for representatives of the y^+^ system. For example, the first characterized murine CAT transports Cys and homoserine only in the presence of Na^+^ (ref. 41). Heterologous expressed human CAT-3 has been shown to mediate Na^+^ as well as K^+^ conductance^42^. Na^+^ dependency has also been reported in some HAT-SLC7 transporters resembling the y^+^L transport system^32^.

In this work, we have achieved unprecedented resolution for electrophysiological characterization of a CAT mechanism and tested a more comprehensive set of substrates compared with previous reports. Hence, it is possible that previous electrophysiological characterization of CAT mechanisms were simply incomplete. Alternatively, it is possible that a majority of vertebrate CATs substantially deviate from the insect *slif* type mechanism in such a way that these transporters either were never Na^+^ coupled or secondarily lost the Na^+^ coupling. Such a possibility received indirect support from the comparative analysis of SBMs, which showed additional negatively charged glutamic acid residues in the AA position 121 (Fig. 1). Such negatively charged SBMs are typical for Na^+^-coupled transport mechanisms. Additional analysis of the *Aa*Slif mechanism could gain important insights about the molecular evolution and structural adaptations of CATs.

AAs play an important role as signalling molecules in mosquito vitellogenesis. Rising levels after a BM activate the TOR signalling pathway in GT, FB and OVs promoting tissue-specific reactions^8^,^10^,^43^,^44^,^45^,^46^. TOR signalling in other key tissues involved in vitellogenesis like nervous system and MTs has not yet been examined but it seems likely that these tissues are able to receive and process AA signals, too.

The *Aa*Slif gene showed tissue-specific expression that was upregulated by BM with 12 and 72 h spikes in several of the tissues we examined (Fig. 5). The upregulation of *Aa*Slif at 12 h precedes the peak of digestive enzyme activities in the GT and the peak in AA concentration in the *Ae. aegypti* haemolymph that coincide between 24 and 30 h PBM^19^. This suggests that female mosquitoes turn up the *Aa*Slif mechanism to achieve rapid directed delivery of AAs to FB and OVs in times of abundance (PBM) and also to balance sufficient intracellular levels of AAs during limited accessibility of these substrates when haemolymph AA levels decline. The rather modest upregulation of AaSlif in the GT PBM is surprising and deserves additional studies. A possible explanation is that *Aa*Slif may act through its Na^+^-independent high-throughput mode at this time in this tissue. Alternatively, the GT may utilize another group of AA transport systems than FB or OVs to shuttle the large amounts of AAs into the haemolymph during this particular time point.

The high AaSlif expression in all tissues we found at 72 h PBM is surprising because at this time the digestion of the BM is already at its end and AA transport between tissues is thought to be declining. We have earlier shown similar transcription upregulation during late vitellogenesis for several other SLC7-type AA transporters, specifically from the HAT family^21^. The gradual reduction of free AA levels at this time might trigger the overexpression of these transporters. In the case of *Aa*Slif, this could compensate for a substrate concentration-dependent switch from its fast low-affinity transport mode to the slow high-affinity mode. An alternative explanation is that these proteins are synthesized in order to allow a quick response to the rapid changes in AA concentrations associated with a second BM. To fully understand the biological relevance of this late upregulation phenomenon it would be interesting to determine the changes in the metabolomes and transportomes in these tissues over the cause of vitellogenesis with a comprehensive transcriptomics/metabolomics survey.

The *Aa*Slif properties and expression profile also support its contribution to AAs signalling in the alimentary and reproductive tissues. Such a contribution is evident from our previous evaluation of the role of CATs in mosquito reproduction^21^,^22^. This is also supported indirectly by data from studies of the *slif* mechanism in *Drosophila*. FB-specific suppression of *Dm*Slif resulted in a global growth defect, similar to that seen in larvae grown under low-nutrient conditions^24^,^47^. *Slif* knockdown in the dopaminergic neurons of the *Drosophila* brain resulted in a strong inhibition of feeding^25^. This phenotype was associated with the General Control Nonderepressing 2-mediated neuronal detection and behavioural rejection of AA-imbalanced meals by fly larvae. Therefore, slif orthologues play an important role in the modulation of insect appetite and dietary source preferences.

An interesting question that warrants further research is to what degree AaSlif is involved in the NO signalling pathway in the mosquito FB. NO is an important activator of the insect innate immune system and involved in the control of bacterial and protozoan parasites^48^,^49^. Nitric oxide synthase catalyzes the synthesis of NO and the byproduct L-citrullin from its substrate L-Arg^50^. Both, L-citrullin and L-Arg are also substrates of AaSlif. Therefore, it is possible if not likely that AaSlif plays a part in the control of NO synthesis by regulating intracellular L-Arg levels and thereby controlling NO synthase activity with consequences for the insect immune system and mosquito vectorial capacity.

*Aa*Slif is the first characterized metazoan transporter with dual affinity for cationic and neutral AAs with a Na^+^-dependent component. Based on its unique properties and strong evolutionary conservation, we propose to define insect-specific orthologues of *Aa*Slif as a new AA transport system, SLIF.

Expression profiles and transport kinetics suggest that *Aa*Slif functions as a high-efficiency transporter genetically and biophysically adapted to act in diverse habitats where unautogenous mosquitoes experience extremely variable availability of nutrient AAs, ranging from ‘very limited’ during a BM-preceding period to ‘over-saturated’ after a BM.

Arthropod-vectored infectious diseases put billions of people at risk worldwide. Novel means to control vector and pests populations are urgently needed. Our study reveals a new insect-specific transceptor for essential AAs, thereby opening new venues for the development of selective and environmentally safe control methods targeting appetite, development and reproduction in pest and vector insects.

## Methods

### Experimental animals

*Ae. aegypti* (Rockefeller strain) was used in all experiments. The mosquito population was maintained in the insectary of the Molecular Disease Vector Physiology Laboratory at New Mexico State University. Mosquitoes with high nutrient reserves were used for all experiments^51^. Larvae were reared using ‘Special Kitty’ cat food (Walmart). Adults were housed in cube-shaped cage (30 cm each side) with 20% sucrose solution at 26.5 °C, 70% RH and 16:8 h (light/dark) cycle. Chickens (*Gallus gallus domesticus*) were used to feed adult female mosquitoes, following the guidelines of the Institutional Animal Care and Use Committee (IACUC) of the New Mexico State University under approved protocol (#2011-041).

### Bioinformatic analysis

For combined phylogenomic-structural analysis, the NCBI protein databases were screened for slif homologues using BLASTp. A set of protein sequences that were considered important and sufficient for comparative analysis of structural and evolutionary aspects of SLC7-CAT mechanisms was selected. Insect genomes were selected based on their taxonomic reciprocity, as well as quality and completes of an existed annotations. The protein sequences undergone multiple sequence alignment with MEGA^52^ implementation of the MUSCLE algorithm^53^ followed by manual correction. The transmembrane helices, known mutation-sensitive site and substrate interaction sites were interpolated from a consensus alignment of selected CATs with crystallized prokaryotic relative from *Methanocaldococcus jannaschii*, MjApcT^27^ and *Escherichia coli*, EcAdiC^28^ (Supplementary Fig. 1B). The TMDs 11–14 were defined by using a TMD prediction algorithm and manual improvement. The coordinate file of bacterial proteins structure (3GIA, 3OB6 and 3L1L) was also used to build 3D models of AeCAT3 in L-arginine occlusion and outside open conformation that was used for docking of L-arginine and identification of additional substrate interaction residues integrated in the final Figures. The Yasara^54^ structure software and MOE LigX^55^ algorithm were used for reconstruction of the 3D model and substrate interaction pattern, respectively. The identified residues position were trimmed from the complete alignment and used for preparing of SBMs diagram using Generous 6 software package^56^.

The evolutionary history was analysed using the neighbour-joining, minimum evolution, maximum parsimony and Bayesian methods that produced similar tree topologies. The neighbour-joining tree was selected as a more common representation of the evolutionary history of transport proteins. The tree was visualized using FigTree 1.3.1 software (A. Rambaut). It was drawn with estimated branch lengths used to infer the tree relative to a 3.7-billion-year-long scale from the root.

### Molecular cloning and RACE verification

Total RNA was isolated from female mosquito 12 h after blood feeding using Trizol (Life Technologies). First-strand cDNA was synthesized on 1 μg of total RNA using Omniscript RT kit (QIAGEN). The *Aa*CAT3 ORF was PCR amplified based on the predicted ORF of AAEL012131-RA gene (VectorBASE) using primers containing *Bam*HI site for upstream primer or *Eco*RI site for downstream primer, cloned into pCR2.1 TOPO (Life Technologies) and sequenced. The *Aa*slif (*Aa*CAT3) ORF was subcloned into an expression vector pXOOM. As we observed low expression signal with this construct, we tested for a possibility of alternative 5′ extension of *Aa*slif ORF in mosquito genome using 5′ RACE. cDNA was synthesized using SMARTer RACE Amplification Kit (Clonetech). One microgram of RNA isolated from 12 h PBM females was used for the synthesis with 5′-CDS primer A and followed the product manual.

Gene-specific RACE PCR and nested PCR primers were designed using the 5′ sequence of AAEL012131-RA (VectorBase, for sequences see Supplementary Table 1). RACE PCR was performed with a gene-specific primer and Universal Primer A Mix or Nested Universal Primer A. RACE PCR products were separated by agarose gel electrophoresis, and bands were excised for gel extraction by QIAGEN Gel Extraction kit (QIAGEN). Gel-extracted PCR products were cloned into pCR2.1 TOPO vector using TOPO TA Cloning Kit (Life Technologies). Resulted 5′ end sequence was used as a query for local BLASTn against *Ae. aegypti* genome sequence (*Aa*egL2, VectorBase) to find the genomic locus, and exon–intron boundary was manually adjusted so that the intron contains canonical splice site (GT-AG).

As we did not identify alternatively spliced variant, we concluded that the low expression was due to suboptimal translation in heterologous system and subsequently optimized *Aa*Slif codon usage for expression in *X. laevis* oocyte. Specifically, the *Xenopus*-optimized *Aa*Slif was synthesized using GENEWIZ service. We also made a C-terminus fusion of *Aa*Slif with eGFP reporter for monitoring the levels of expression and appropriate trafficking of the *Aa*Slif in individual oocytes. The *Aa*Slif (with or without eGFP) sequence was PCR amplified and cloned into pCR2.1 TOPO (Life Technologies) and restriction-digested insert was subcloned into the expression vector pXOOM using restriction sites *Bam*HI (on 5′) and *Not*I (on 3′).

### Heterologous expression and characterization

cRNA for heterologous expression was synthesized by *in vitro* transcription of *Xba*I-linearized *Aa*slifco-pXOOM and *Aa*SlifcoeGFP-pXOOM plasmids using the mMessage-mMachine (Ambion Inc.). The integrity and quantity of the RNA was confirmed by agarose gel electrophoresis. Surgically isolated, collagenase-treated and defolliculated stage V–VI *X. laevis* oocytes were purchased from Ecocyte Bioscience US LLC and injected with ∼35 ng of *Aa*Slif cRNA using Nanoliter 2000 injector (WPI). Oocytes were incubated for 2–6 days at 17 °C in sterile 98 mM Na^+^ oocyte media supplemented with 2.5 mM sodium pyruvate, 100 U ml^−1^ penicillin, 100 mg ml^−1^ streptomycin and 5% horse serum. For ion dependency assay, the 98 mM Na^+^ was substituted with the equimolar amount of K^+^, Li^+^ or NMDG^+^ . The exact recipes for solution preparation are summarized in the Supplementary Table 1. The substrate-induced currents were recorded from voltage clamped oocytes at constant flow perfusion. The holding voltage was −50 mV, except for ramp stimulation. The *I*/*V* data sets were acquired during ramp stimulation (step −130 mV; 200 ms, ramp −130 to +40 mV in 1 s; Supplementary Fig. 2A). The *I*/*V* recording (1 kHz sampling) was filtered with digital 50 Hz low pass 8 pole Bessel filter, reduced by factor 30 (substitute average) and used to build *I*/*V* plots. The *I*/*V* before substrate application was subtracted to eliminate substrate-independent current component. Additional corrections include subtraction of baseline drift current, which reflects slow adaptation of ion pumps after ion substitution (typically <5 nA), if required. The data (mean±s.d., *n*>2 oocyte/sample for each point) were fit with a preferable two-site specific binding model: Site1=BmaxHi*X/(*K*_d_^Hi^+*X*), Site2=BmaxLo*X/(*K*_d_^Lo^+*X*) and *Y*=Site1+Site2, where Bmax is apparent saturation current for high- and low-affinity components, respectively, *X* are actual current values and *K*_d_ are apparent dissociation constants. One site-specific binding with Hill slope model was used as an Null hypothesis in evaluation of organic substrate-binding kinetic and for approximation of Na^+^ binding data: *Y*=Bmax*Xη /(*K*_d_*η*+*Xη*), where *η* is apparent Hill constant.

### Radiolabelled amino-acid uptake assay

*Aa*Slif-expressing and control oocytes were conditioned in AA-free ND98 for 3–4 h and placed in 600 μl of 98 Na^+^ media containing 2 mM of final concentration of L-Arg and L-Lys with added 10 μl of 0.1 μCi μl^−1^ of L-[14C(U)]-Arg or 1 μCi μl^−1^ of L-[4,5-3H(N)]-Lys, respectively. After incubation for 20 min at room temperature, oocytes were individually rinsed twice with excessive volume of ice-cold 98 Na^+^ media and lysed in 0.2 ml of 1% SDS solution with ultrasonic treatment. The individual samples were diluted with 2 ml of scintillation liquid and radioactivity was measured using a Beckman-Coulter LS6500 scintillation counter (Beckman). The AA uptake rate was extrapolated considering the isotope-labelled amounts, final concentrations and specific activities of AAs (SA: L-Arg, 0.31 and L-Lys, 45 Ci mmol^−1^, Moravek Biochemicals). Disintegration per minute (DPM) data were converted to mmol min^−1^ using a constant (1 Ci=2.22 × 1012 DPM): mmol min^−1^=(DPM × molar ratio [all/isotope])/(2.2 × 1012 × SA × 20). Final data are shown in pmol min^−1^.

### Statistical analysis

Each experiment was repeated at least three times, using oocyte batches from different isolations. Statistical significance in the electrophysiology experiments was determined by unpaired Student’s *t*-test. All underlying assumptions of the test were met by the data. For quantitative PCR, triplicate samples prepared from different mosquito individuals were analyzed. Two-way analysis of variance followed by Tukey’s HSD *post-hoc* test was used to validate significant differences of a spatio-temporal gene expression. The data met the assumptions of the test. The PRIZM5 (GraphPad Software, Inc.) and SigmaPlot/SigmaStat (Systat Software, Inc.) software was used for data collection, statistical evaluation and final graph preparation. Statistical significance values and analysis-specific details are included in the result section and figure legends.

### qPCR tissue expression analysis

RNA isolation, cDNA synthesis and qPCR analysis were completed in compliance with the MIQE guidelines^57^. Primer BLAST (http://www.ncbi.nlm.nih.gov/tools/primer-blast/) was used to develop gene-specific primers (Supplementary Table 2). Whole female, FB, thorax, MT, OVs and GT were dissected from non-blood fed, 3, 12, 24, 48, 72 and 96 h post BM adult females. RNA was isolated using Trizol (Invitrogen). First-strand cDNA synthesis was performed on 1 μg of total RNA in 20 μl reaction using Qiagen Omniscript RT Kit (Qiagen). Transcripts were normalized by qPCR analysis of ribosomal protein S7 (rpS7) levels(49) on an Eppendorf Mastercycler ep realplex (Eppendorf) using iQ Supermix (Bio-Rad) with 25 μl volume reactions. Each experiment was done with three independent biological replicates. PCR conditions were as follows: an initial incubation at 95 °C for 2 min; followed by 40 cycles of 95 °C for 15 s, 55 °C for 15 s, 72 °C for 20 s.

## Additional information

**How to cite this article:** Boudko, D. Y. *et al*. Substrate specificity and transport mechanism of amino acid transceptor Slimfast from *Aedes aegypti*. *Nat. Commun.* 6:8546 doi: 10.1038/ncomms9546 (2015).

## Supplementary Material

Supplementary Information Supplementary Figures 1-4, Supplementary Tables 1-2 and Supplementary References
