# Supplementary material for: Substrate specificity and transport mechanism of amino-acid transceptor Slimfast from Aedes aegypti
Source: Nat Commun. 2015 Oct 9;6:8546. doi: 10.1038/ncomms9546 (PMC4608377; doi:10.1038/ncomms9546)
Supplement: Supplementary Information — Supplementary Figures 1-4, Supplementary Tables 1-2 and Supplementary References [file ncomms9546-s1.pdf]

Supplemental Material

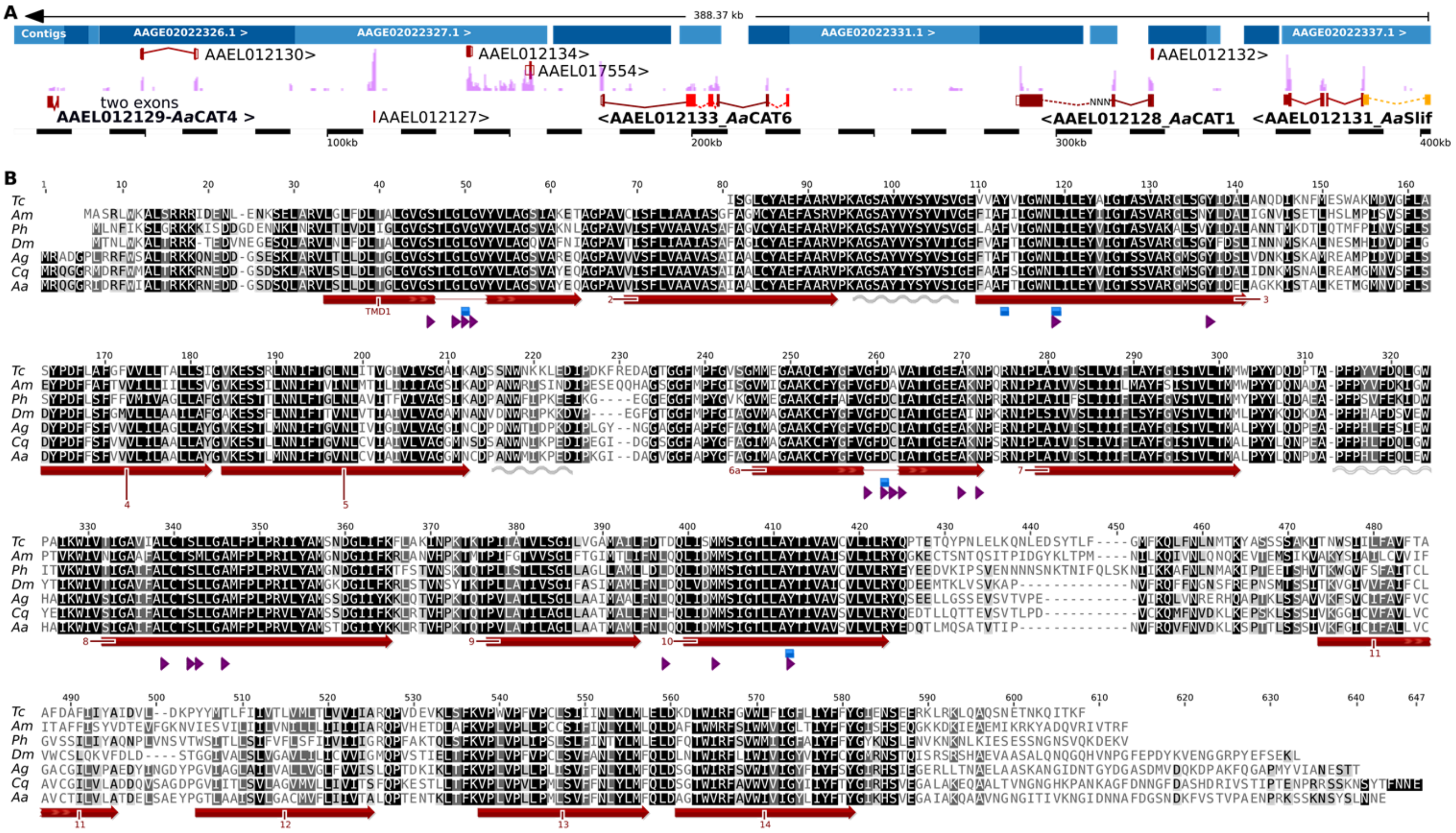

Supplementary Figure 1 A. Structure of the *Aa*Slif gene and associated gene cluster in the *Ae. aegypti* genome. Experimentaly validated splicing of the *Aa*Slif gene from this study is shown as orange-colored shapes (dotted lines for intron and box for exons). Corrected splicing of previously cloned *Aa*CAT1 cannot be shown in the figure as additional part is in a "gap (a strech of Ns)" in the genome (shown as "NNN"). The corrected splicing of predicted *Aa*CAT2 are shown as red shapes. Direction of the genes is shown as > and < symbols in the gene annotation. The magenta pattern represents a relative gene expression ratio in a mixed sex whole animal sample as retrived from the current VectorBase data set after validation by new generation transcriptome sequencing (1). **B. A sequence alignment of orthologous Slif proteins from different insect species.** Transmembrane domains (TMD#, red arrow), substrate binding motif (magenta arrowhead), and known mutation sensitive site (blue square) were extrapolated from a consensus alignment of selected CATs with crystallized prokaryotic relative, *Methanocaldococcus jannaschii* *Mj*ApcT (2) and *Escherichia coli* *Ec*AdiC (3), except for TMD11-14 that were defined by using a TMD prediction algorithm. The *slif* orthologs from *Tribolium castaneum* (Tc) was partially predicted due to incomplete upstream sequence in the genomic scaffold. The *Anopheles gambiae* (*Ag*) *slif* ortholog was partly annotated in VectorBase as two proteins (AGAP010560 and AGAP010561) and was defined as splicing domains of a single protein here using FGENESH+ (Softberry.com). The plausible translation of the predicted gen is included in the alignment.

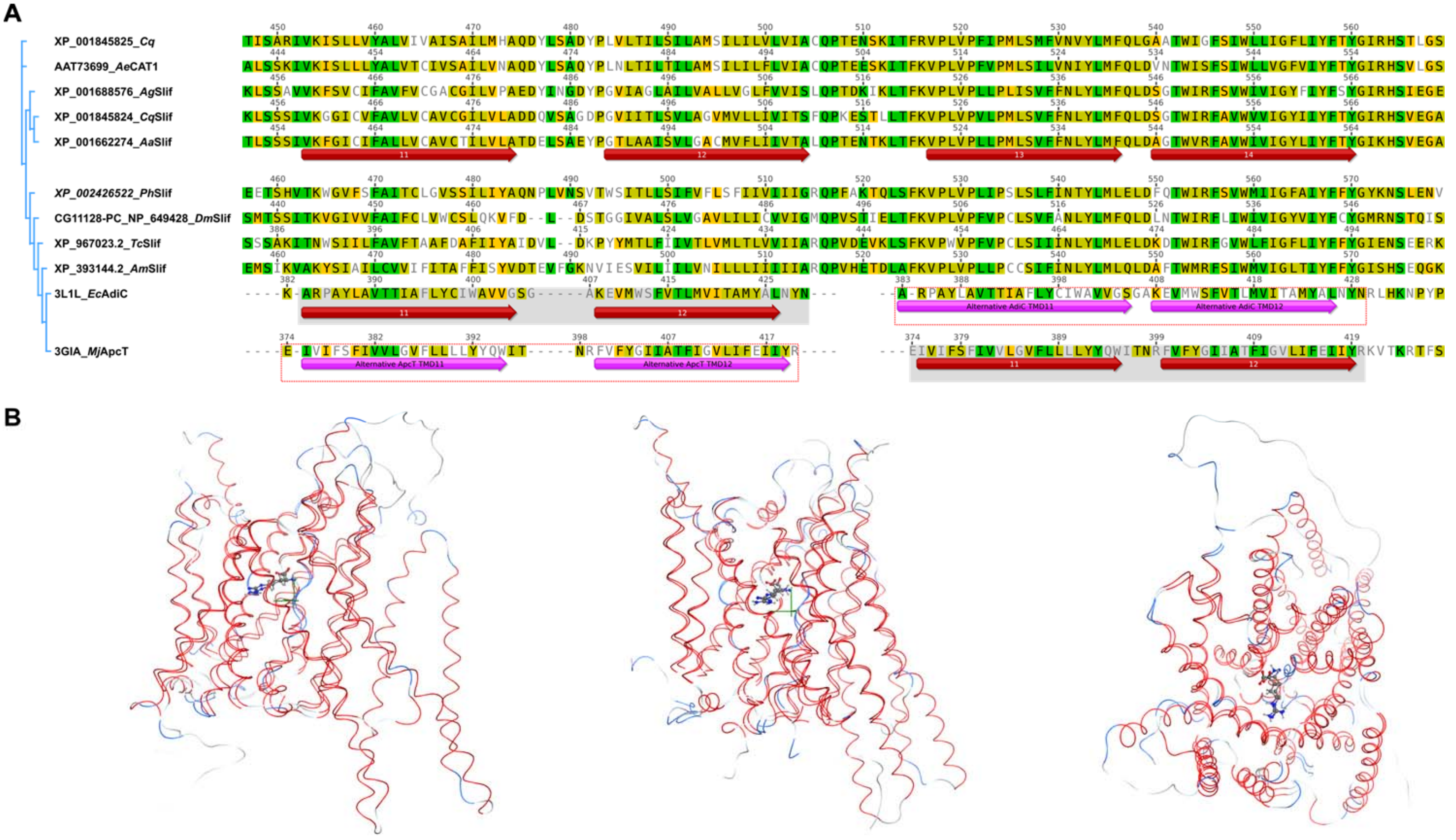

Supplementary Figure 2. Structural aspect of *Aa*Slif and CATs. A. Alignment of insect CATs TMDs 11-12 and TMDs 13-14 pairs with TMDs 11-12 of crystalized bacterial homologs, *Ec*AdiC and *Mj*ApcT. Magenta TMD arrows indicate TMDs 11-12 of *Ec*AdiC and *Mj*ApcT. The red TMD arrows and gray background indicated preferable aliment. The background color code for sequence similarity is based on the Blosum 45 AA similarity matrix with a -1 threshold: green 100%, salad 80% orange 60% and blank < 60% of similarity. **B.** Structural aliments of a 3D homology model of the *Aa*Slif with the 3L1L structure of *Ec*AdiC. 0, 90 degree orthogonal and tope view projections are shown with L-Arg binding in the occlusion site.

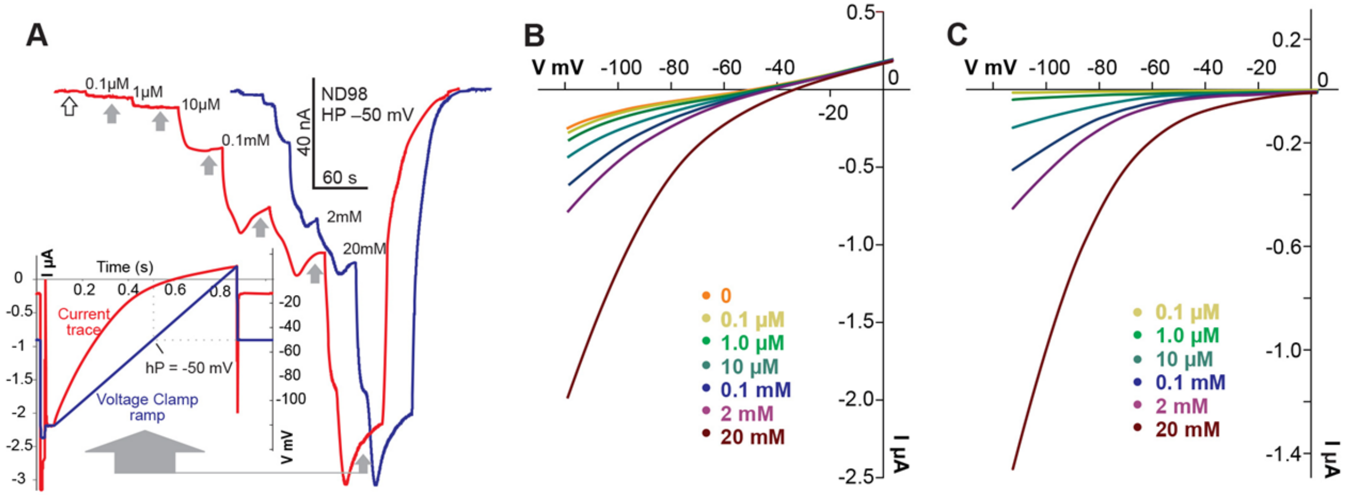

Supplementary Figure 3. *Aa*Slif substrate saturation kinetic and I/V plot. A. Saturations kinetic of L-Arg transport is demonstrated as current traces recorded from *Aa*Slif (red trace) and *Aa*Slif-eGFP fusion (blue trace) expressing oocytes. L-Arg was applied in an increment staircase concentration trend as labeled on the panel. The arrows indicate points where voltage ramp was triggered to obtain current (I) and voltage (V) profiles as shown on insert (the short large-amplitude stimulation pikes were removed from the red current traces for clarity). Lower-left traces show a representative voltage ramp recording (red, current trace-left y-axis; blue, voltage trace-right y-axis). B. Superposed I/V plots at different concentration of L-Arg built from the ramp recorded in points indicated by arrows at panel A. C. I/V after subtraction of a leak current trace (orange trace on B), recorded at the point indicated by empty arrow on panel A.

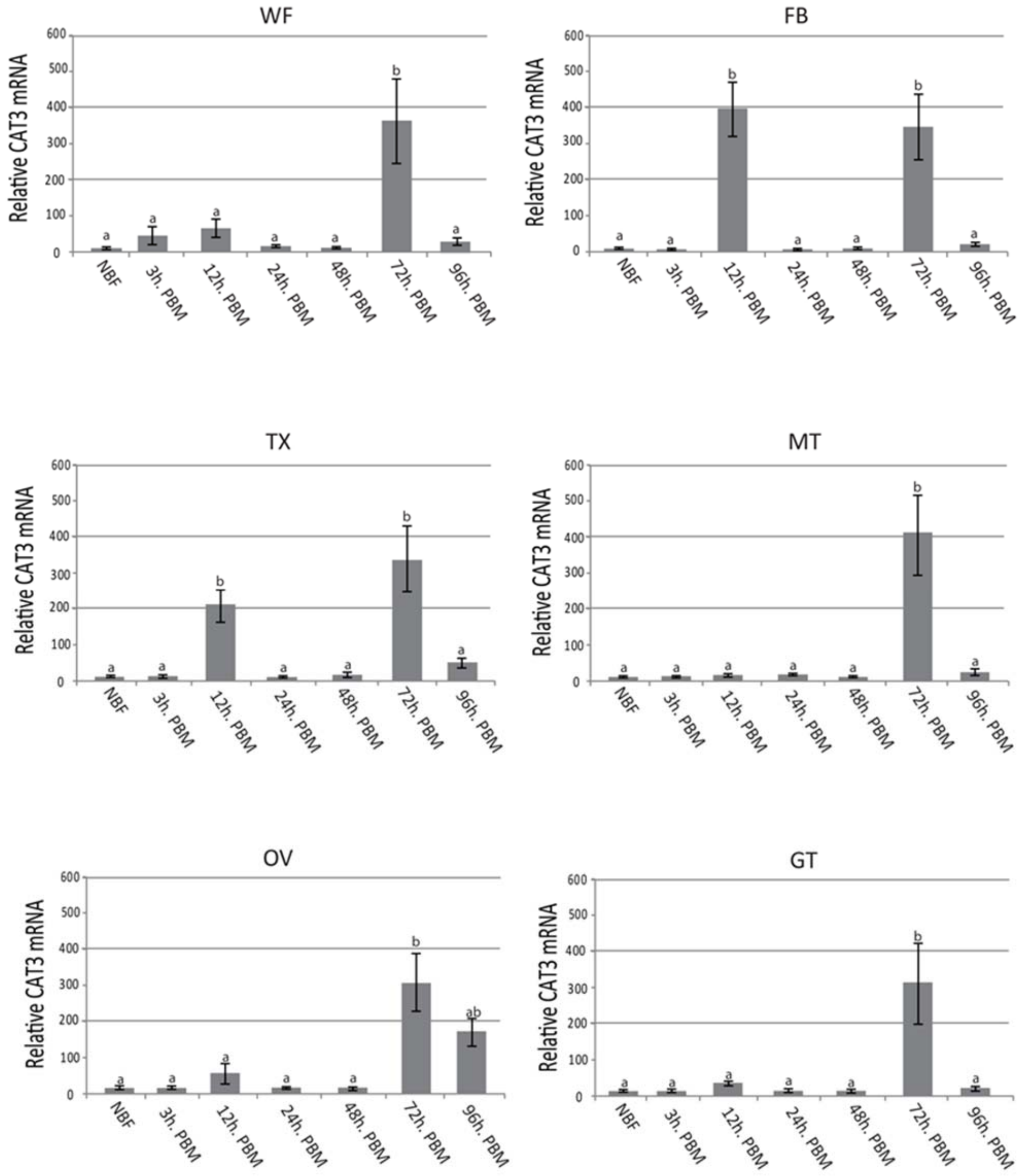

Supplementary Figure 4. *Aedes aegypti* CAT3 expression in various tissues/organs. Expression was assayed using qPCR. The data represent relative quantification of Aedes AQPs which were normalized by qPCR analysis of ribosomal protein S7 (rpS7) mRNA levels in the cDNA samples. Values are means ± S.E. (error bars) of triplicate biological samples with similar results. Means separated by Tukey-Kramer HSD ($p<0.05$). Means which share the same letter are not significantly different. RNA was isolated from whole body and various tissues/organs of adult female mosquitoes non-blood fed (NBF), 3h, 12h, 24h, 48h, 72h, and 96h post-blood meal (PBM). WF-Whole female, FB-fatbody, TX-thorax, MT – Malpighian tubules, OV – ovaries, GT-midgut.

Supplementary Table 1. Recipes of the buffer solutions used for electrophysiological characterization of the *Aa*Slif mechanism

Buffer #		#1<br>98 Na^+		#2<br>98 K^+		#3<br>98 $NMDG^+$		#4<br>98 Li^+	
**Salt**	mol wt	mM	g/l	mM	g/l	mM	g/l	mM	g/l
NaCl	58.44	**98**	5.7	**1.0**					
KCl	74.55	**2.0**		**98**	7.3				
NMDG	195.2					**98**	19.1		
LiCl	24.4							**98**	4.2
$MgCl_2\ 6H_2O$	203	**0.5**	0.1	**0.5**	0.1	**0.5**	0.1	**0.5**	0.1
$CaCl_2\ 2H_2O$	147.0	**0.5**	0.1	**0.5**	0.1	**0.5**	0.1	**0.5**	0.1
HEPES	238.3	**10**	2.4	**10**	2.4	**10**	2.4	**10**	2.4
pH<br>(adjust)		7.2<br>(4N NaOH)		7.2<br>(4N KOH)		7.2<br>(4N HCl)		7.2<br>(4N LiCl)	

Supplementary Table 2. Primer list used in the study

Name	Use	Sequence 5'-3'
GSPCAT3R1	5'RACE	CCCACCCCCAGTCCGGTCAGATCC
GSPCAT3R2	nested 5'RACE	CCAGTCCGGTCAGATCCAGCAGCGA
GSPCAT3R3	nested 5'RACE	CCGGTCAGATCCAGCAGCGAGAGGAC
CAT3F	initial expression	GGATCCATGATGCGCCAAGGTGGCCGGATAGACCGT
CAT3R	initial expression	GAATTCTTCATTGTTGAGTGAGTACGAATTCTTGGAACTCTTCCGGGGAT
CAT3XF_NB	codon-optimized subcloning	GCTAGCGGATCCACCATGCGTCAGG
CAT3XR_NM	codon-optimized subcloning	ACGCGTGCGGCCGCTTACTCGTTGTTCAGAGAGTA
CATXGR_NM	codon-optimized subcloning (C-term eGFP)	ACGCGTGCGGCCGCTCACTTGTACAGCTCGTC
AaCAT3F	qPCR	AACGTGGGTCCGATTCGCCG
AaCAT3R	qPCR	TCTCGGCCGGGACTGTCGAT

Relevant restriction sites are indicated by underline.

Supplementary References

1. Gibbons JG, *et al.* (2009) Benchmarking next-generation transcriptome sequencing for functional and evolutionary genomics. *Mol Biol Evol* 26(12):2731-2744.
2. Shaffer PL, Goehring A, Shankaranarayanan A, & Gouaux E (2009) Structure and mechanism of a Na+-independent amino acid transporter. (Translated from eng) *Science* 325(5943):1010-1014 (in eng).
3. Gao X, *et al.* (2010) Mechanism of substrate recognition and transport by an amino acid antiporter. (Translated from eng) *Nature* 463(7282):828-832 (in eng).
4. Tamura K, *et al.* (2011) MEGA5: molecular evolutionary genetics analysis using maximum likelihood, evolutionary distance, and maximum parsimony methods. (Translated from eng) *Molecular biology and evolution* 28(10):2731-2739 (in eng).
5. Edgar RC (2004) MUSCLE: multiple sequence alignment with high accuracy and high throughput. (Translated from eng) *Nucleic acids research* 32(5):1792-1797 (in eng).
6. Krieger E, Koraimann G, & Vriend G (2002) Increasing the precision of comparative models with YASARA NOVA--a self-parameterizing force field. (Translated from eng) *Proteins* 47(3):393-402 (in eng).
7. Inc. CCG (2015) Molecular Operating Environment (MOE), 2013.08.
8. Kearse M, *et al.* (2012) Geneious Basic: an integrated and extendable desktop software platform for the organization and analysis of sequence data. *Bioinformatics* 28(12):1647-1649.
9. Huggett JF, *et al.* (2013) The digital MIQE guidelines: Minimum Information for Publication of Quantitative Digital PCR Experiments. *Clin Chem* 59(6):892-902.
10. Anonymous (2010) Primer BLAST.
